# Abnormal Serum Free Light Chain Ratios in the Absence of Clonal Plasma Cell Disease: A Review of the Literature

**DOI:** 10.7759/cureus.117423

**Published:** 2026-10-04

**Authors:** Akhil Jose, Mohammed Mounuddin, Mudit Bhatnagar, Sri Krushna Prasad Mishra, Gaurav Borse, Ambili Remesh, Abdul M Barbhuiya, Neena Bhatti

**Affiliations:** 1 Orthopedics and Traumatology, KMCT Medical College, Kozhikode, IND; 2 Biochemistry, Raja Rajeshwari Institute of Medical Sciences - Girls, Sangareddy, IND; 3 Pharmacology, Sukh Sagar Medical College and Hospital, Jabalpur, IND; 4 General Medicine, Sarada Nursing Home, Puri, IND; 5 Pharmacology, Poona College of Pharmacy, Pune, IND; 6 Pharmacology and Therapeutics, Sree Uthradom Thiurnal Academy of Medical Sciences, Thiruvananthapuram, IND; 7 Pharmacology, Nemcare Group of Institutions, Guwahati, IND; 8 Pharmacology and Therapeutics, Christian Medical College and Hospital, Ludhiana, IND

**Keywords:** chronic kidney disease, kappa/lambda ratio, laboratory medicine, light-chain mgus, polyclonal free light chains, reference interval, serum free light chain

## Abstract

The serum free light chain (sFLC) κ/λ ratio is an important component of laboratory evaluation of plasma cell disorders and is incorporated into the diagnostic assessment of light-chain monoclonal gammopathy of undetermined significance and risk stratification of monoclonal gammopathies. Its usefulness rests on an assumption that deserves closer scrutiny: that a ratio falling outside the conventional diagnostic interval of 0.26-1.65 signifies clonality. This review examines the literature published between June 2016 and June 2026 on the frequency of abnormal κ/λ ratios in individuals without clonal plasma cell disease and the factors that contribute to these findings. The frequency of an abnormal ratio varies substantially according to the population studied, renal function, and the reference interval applied. Use of age-stratified, estimated glomerular filtration rate-banded, or locally derived reference intervals generally reduces the proportion of individuals classified as having an abnormal ratio. Important determinants include reduced glomerular filtration, assay-platform and calibrator variation, advancing age, and polyclonal immune activation associated with autoimmune disease, infection, and critical illness. Elevated concentrations of polyclonal free light chains may also have prognostic associations, while an isolated abnormal ratio may not be reproducible over time. Interpretation of the κ/λ ratio should therefore consider age, renal function, assay characteristics, and the clinical context, with orthogonal laboratory assessment used when appropriate before assigning a plasma cell precursor diagnosis.

## Introduction and background

Immunoglobulin light chains are synthesized by plasma cells in slight excess of heavy chains, and the surplus is released into the circulation as free κ and free λ. Because these small proteins are cleared almost entirely by glomerular filtration and proximal tubular reabsorption, their serum concentrations reflect the balance between plasma cell output and renal handling [1,2]. Quantification of serum free light chains (sFLCs) by immunoassay transformed the laboratory assessment of plasma cell disorders, replacing much of the reliance on urine studies and providing a sensitive route to the detection of light-chain-only clones [1,3].

The clinical value of the assay rests less on the absolute concentrations than on the κ/λ ratio. Because a clone produces one light-chain isotype only, a skewed ratio is taken as evidence of clonality, whereas parallel elevation of both isotypes is read as polyclonal. On this logic, the ratio is incorporated into the diagnostic assessment of light-chain monoclonal gammopathy of undetermined significance (LC-MGUS) and into risk stratification and diagnostic evaluation across several plasma cell disorders, and in the risk models that determine how intensively a person with a precursor state is investigated and followed [4,5].

By current International Myeloma Working Group criteria, an abnormal κ/λ ratio alone does not establish LC-MGUS. LC-MGUS requires an abnormal free light-chain ratio with an increased concentration of the involved free light chain, absence of an intact immunoglobulin heavy chain on immunofixation, a clonal bone-marrow plasma-cell burden below 10%, urinary monoclonal protein below 500 mg/24 hours, and absence of myeloma-related end-organ damage [4,6]. Conventional MGUS and LC-MGUS should therefore be distinguished when interpreting studies of the diagnostic performance of the sFLC ratio. In particular, an abnormal ratio is not present in all cases of conventional MGUS, and findings from general MGUS cohorts should not be interpreted as direct estimates of the sensitivity or specificity of the ratio for LC-MGUS. The Shenzhen screening study illustrates this distinction: it evaluated MGUS in a general health-check population, and only 31.1% of confirmed MGUS cases had an abnormal sFLC ratio [7]. This finding demonstrates the imperfect concordance between the ratio and MGUS in an unselected population rather than establishing the diagnostic performance of the ratio specifically for LC-MGUS.

That convention assumes the reference interval is fit for purpose. The widely used diagnostic range of 0.26-1.65 was derived from a modest cohort of healthy donors and does not obviously translate to the population in which the test is now ordered - an older, frequently renally impaired, frequently inflamed clinical population. Two developments have made this assumption difficult to sustain. First, large population screening studies have shown that a substantial minority of people with no clonal disease at all fall outside the standard interval, and that revised age-stratified intervals can reclassify most of them as normal without missing progression events [6]. Second, kidney function alone shifts the ratio enough to reclassify a tenth of a chronic kidney disease (CKD) cohort [8].

As arguments for population-based screening for myeloma precursors gather momentum, the specificity of the ratio in unselected people becomes a question of public-health importance rather than laboratory housekeeping [9]. The stakes are not trivial. A spuriously abnormal ratio may trigger bone marrow sampling, skeletal imaging, indefinite hematology follow-up, and the psychological burden of a cancer precursor label; the same result may also prompt an unnecessary kidney biopsy when monoclonal gammopathy of renal significance is suspected [10,11]. Conversely, uncritical dismissal of an abnormal ratio risks missing genuine light-chain-restricted disease.

Despite a large primary literature, the frequency with which an abnormal sFLC ratio occurs in people without clonal plasma cell disease, and the relative contribution of renal, analytical, demographic and immunological factors to that abnormality, have not been brought together in one place. This review of the literature sets out the published evidence on both questions, examines the mechanisms that link each determinant to the observed shift in the ratio, and considers what follows for laboratory reporting and bedside interpretation.

## Review

Scope and approach to the literature

The literature discussed here was identified through searches of MEDLINE via PubMed, Embase, Scopus, Web of Science Core Collection and the Cochrane Library covering English-language articles published between 1 June 2016 and 30 June 2026 (databases last searched on 30 June 2026), supplemented by hand-searching of the reference lists of retrieved articles and of relevant narrative reviews. Search terms combined three concept blocks: the analyte ("free light chain", "kappa lambda ratio", "Freelite", "N Latex", "sFLC"); the measurement construct ("reference interval", "reference range", "diagnostic range", "prevalence", "false positive"); and the population ("general population", "healthy", "screening", "chronic kidney disease", "autoimmune", "non-clonal").

Preference was given to reports in which clonal plasma cell disease had been explicitly excluded, or in which monoclonal cases were separately identified and removed, and in which the reference or diagnostic interval applied was stated, since only such studies allow an abnormal ratio to be attributed to something other than a clone. Population screening programs, cross-sectional and cohort studies, reference-interval and method-comparison studies, and quantitative syntheses all contribute. Cohorts composed exclusively of patients with established multiple myeloma or light-chain amyloidosis are drawn on only where they illuminate a specific determinant.

This review was designed as a narrative synthesis rather than a systematic review. Literature was purposively selected to provide a clinically focused synthesis of heterogeneous evidence relevant to non-clonal abnormalities of the sFLC κ/λ ratio. Because the review was not designed as a systematic evidence synthesis, formal PRISMA reporting, quantitative risk-of-bias assessment, and exhaustive accounting of screened and excluded records were not undertaken. Studies addressing MGUS prevalence, environmental exposures, obesity, or progression were included selectively when they provided relevant contextual information for factors discussed in relation to sFLC interpretation. The principal studies drawn upon are summarized in Table 1 [6,8,12-33]. 

**Table 1:** Principal studies informing this review ATTR, transthyretin amyloidosis; ATTRwt, wild-type transthyretin amyloidosis; CI, confidence interval; CKD, chronic kidney disease; eGFR, estimated glomerular filtration rate; FLC, free light chain; HCV, hepatitis C virus; HR, hazard ratio; ICU, intensive care unit; IFE, immunofixation electrophoresis; iStopMM, Iceland Screens, Treats, or Prevents Multiple Myeloma; LC-MGUS, light-chain monoclonal gammopathy of undetermined significance; MGUS, monoclonal gammopathy of undetermined significance; OR, odds ratio; RA, rheumatoid arthritis; sFLC, serum free light chain; sIFE, serum immunofixation electrophoresis; SLE, systemic lupus erythematosus; SPEP, serum protein electrophoresis; ΣFLC, summed free light chains

Study (year), reference	Country; design	Population (n)	Assay	Reference interval applied	Principal finding relevant to this review
Einarsson Long et al. (2025) [6]	Iceland; prospective population screening (iStopMM)	Adults ≥40 y with preserved kidney function (41,882)	Freelite	0.26-1.65; revised age-stratified 99% intervals	sFLC ratio abnormal in 3.7%; κ abnormal in 17.5% and λ in 4.0%. Revised ratio intervals 0.44-2.16 (<70 y) and 0.46-2.59 (≥70 y). LC-MGUS prevalence fell from 1.54% to 0.27%; none of the 1,006 reclassified individuals progressed over a median 4.6 years.
Long et al. (2022) [8]	Iceland; population screening, CKD subgroup (iStopMM)	Non-dialysis CKD, eGFR <60 mL/min/1.73 m², no monoclonality (6,461)	Freelite	0.26-1.65; renal interval 0.37-3.10	Ratio abnormal in 9.0% by the standard interval versus 0.7% by the renal interval; κ out of range in 60% and λ in 21%. New eGFR-banded 99% intervals proposed (0.46-2.62, 0.48-3.38, 0.54-3.30). Crude LC-MGUS 0.5%.
Luo et al. (2025) [12]	China; multicenter cross-sectional	All-cause non-dialysis CKD (5,287)	Freelite	Standard, renal, iStopMM and locally derived	Ratio abnormal in 10.5% (standard), 0.3% (renal), 0.5% (iStopMM) and 0.9% (locally derived); κ above the standard limit in 89.0% and λ in 72.1%. Crude LC-MGUS fell from 10.4% to 0.9%, and was κ-restricted in 99.8% of cases.
Treger et al. (2024) [13]	USA; retrospective informatics cohort	Consecutive clinical samples without evidence of monoclonality by sFLC assessment and immunofixation (5,057)	Freelite (Optilite)	0.26-1.65; derived 0.67-2.13	25% (1,226/5,057) had a ratio outside the standard interval. The derived interval reduced the apparent-abnormality rate to 8%. Misclassification was not explained by sex, age, kidney function, or antisera lot. A normal ratio persisted in 16% (14/85) of samples with free λ on immunofixation.
Waggoner et al. (2026) [14]	USA; three-center method comparison	Healthy volunteers screened by capillary electrophoresis (124)	Freelite (Optilite)	0.26-1.65	6-7% of healthy participants fell above the diagnostic range with both the prior and the reformulated κ calibrator; the manufacturer's κ reference interval could not be verified with either calibrator.
Azimi et al. (2023) [15]	USA; retrospective data mining and method development	Cohorts spanning the full clinical spectrum of renal function	Freelite (Roche cobas c501)	Manufacturer's versus newly derived	A single renally robust ratio interval and a principal-component-based metric both lowered the apparent-abnormality rate without loss of sensitivity for monoclonal gammopathy.
Griffiths et al. (2024) [16]	Canada; method comparison	Sera with normal and abnormal creatinine (260)	Freelite (Optilite); Diazyme (Architect)	Manufacturer's	At eGFR ≥60 the ratio met the manufacturer's claim in 91% (Freelite) and 95% (Diazyme). With falling eGFR the Freelite ratio rose while the Diazyme ratio fell.
Rocamonde and Hermida (2023) [17]	Spain; reference-interval study	Healthy blood donors (246)	Freelite (Optilite)	Locally derived (CLSI EP28-A3C)	Local diagnostic interval 0.50-2.11, wider than the manufacturer's. Classification was discordant in 14.5% of MGUS and 16.3% of myeloma samples.
Sprangers et al. (2020) [18]	Belgium; cross-sectional	Mild-to-moderate CKD	Freelite; N Latex	Manufacturer's	The Freelite ratio correlated inversely with eGFR whereas the N Latex ratio correlated positively. The two assays are not interchangeable in CKD.
Morales-García et al. (2023) [19]	Spain; retrospective	CKD stages 2-5 (226)	Freelite (Optilite); Kloneus (AU5800)	Manufacturer's	In identical sera the median ratio was 1.50 (range 0.66-3.45) by Freelite and 0.63 (95% range 0.34-1.01) by Kloneus - opposite directions of bias.
Lutteri and Jacobs (2018) [20]	Belgium; reference-range study	Patients with CKD	Sebia FLC	Sebia-specific	The Sebia ratio behaves differently from Freelite across CKD stages; assay-specific renal ranges are required.
Lutteri et al. (2018) [21]	Belgium; method evaluation	Routine, control and CKD sera	Sebia ELISA (AP22 ELITE); Freelite	Manufacturer's	82% concordance with Freelite. κ and λ were both higher in CKD, and a CKD-specific ratio interval was judged necessary for Sebia as well.
Xu L et al. (2022) [22]	China; observational	Adults with CKD, monoclonal gammopathy excluded	Freelite; N Latex	Manufacturer's	The N Latex ratio remained within the healthy reference range irrespective of renal function; the Freelite ratio did not. N Latex FLC tracked β₂-microglobulin closely.
Rauf et al. (2023) [23]	Multinational; retrospective multicenter	Suspected or histologically proven cardiac amyloidosis (3,354)	Freelite	eGFR-refined cut-offs	Refined eGFR-dependent ratio cut-offs improved exclusion of monoclonal gammopathy; non-biopsy criteria for ATTR cardiomyopathy were 97% specific (95% CI 91%-99%).
Geller et al. (2017) [24]	USA; retrospective cohort	Wild-type transthyretin amyloidosis (113)	Freelite	Standard and renal	Monoclonal gammopathy in 23%. A further seven patients (6.2%) had an abnormal ratio attributable to renal dysfunction alone.
Phull et al. (2018) [25]	USA; retrospective cohort	ATTRwt and ATTR V122I amyloidosis (197)	Freelite	Standard	MGUS - defined in part by an abnormal ratio - in 39% of ATTRwt and 49% of ATTR V122I patients, far above age-matched population rates.
Pelzer et al. (2018) [26]	Germany; population-based longitudinal (Heinz Nixdorf Recall)	Adults aged 45-75 y (4,814); 75 with LC-MGUS	Freelite	0.26-1.65	On serial testing 17 of the 31 cases with repeat samples (55%) could not be confirmed, and none of the 75 cases progressed over a median 11.5 years.
Kumar et al. (2019) [27]	USA; population-based cohort (Olmsted County)	Adults without MGUS at baseline (15,630)	Freelite	Standard	The highest decile of polyclonal ΣFLC carried a 2.6-fold (95% CI 1.8-3.7) risk of later monoclonal gammopathy over a median 18.1 years.
Fraser et al. (2017) [28]	International; systematic review with individual-patient data meta-analysis	Five prospective CKD cohorts (3,912)	Freelite	ΣFLC >43.3 mg/L	ΣFLC above the upper limit independently predicted mortality (HR 1.45, 95% CI 1.14-1.85) and end-stage renal disease (HR 3.25, 95% CI 1.32-7.99).
Bettacchioli et al. (2021) [29]	Europe; cross-sectional (PRECISESADS)	Systemic autoimmune disease (1,979) and healthy controls (614)	Turbidimetric	Manufacturer's	Total FLC were raised in 28.4% of patients, with a preserved κ/λ ratio. Elevation tracked disease activity and the type I interferon signature, independent of kidney function.
Gulli et al. (2019) [30]	Italy; cross-sectional	RA, SLE, antiphospholipid syndrome, primary Sjögren syndrome and HCV cryoglobulinemia (260); controls (50)	Turbidimetric	Manufacturer's	κ rose more than λ across every disease group, producing a mean κ/λ of approximately 1.6 - a polyclonal shift toward the upper reference limit.
Shankar-Hari et al. (2017) [31]	UK; prospective cohort	Adults with sepsis and no immune comorbidity (101)	Freelite	Population normal ranges	On ICU day 1, κ was high in 75.3% and λ in 46.5%. Concentrations correlated with the frequency of dual κ/λ-expressing CD19⁺ B cells (4.1% vs 1.3% in healthy controls).
Cicero et al. (2022) [32]	South Africa; cross-sectional screening	Black men aged >30 y (386)	SPEP with FLC quantification	Standard	MGUS prevalence 8.03% (95% CI 5.32-10.74), nearly 1.6-fold higher than in white men in Olmsted County. Current smoking was independently associated with MGUS (adjusted OR 4.49, 95% CI 1.63-14.56); the association with HIV status did not persist after adjustment.
Xu et al. (2024) [7]	China; cross-sectional screening (Shenzhen)	Health-check attendees (49,220)	SPEP/sIFE with FLC quantification	Standard	MGUS prevalence 1.17% (95% CI 1.08-1.27). An abnormal FLC ratio was present in only 31.1% of confirmed MGUS, underlining its imperfect concordance with clonality.
Andersen et al. (2026) [33]	Denmark; nationwide retrospective clinical cohort (external validation)	Registry-identified LC-MGUS cases, DALY-CARE resource (365 total; 215 revised-criteria, 150 reclassified)	Freelite	Original vs revised (iStopMM) criteria	Applying the revised iStopMM criteria reduced LC-MGUS diagnoses by 40% (150/365 reclassified as normal). None of the reclassified individuals progressed to multiple myeloma or AL amyloidosis over a median 3.7 years' follow-up, although two (1.3%) progressed to non-Hodgkin lymphoma. Among those retaining a revised LC-MGUS diagnosis, 9.8% progressed (5.1% to multiple myeloma, 3.3% to AL amyloidosis).

Epidemiology of MGUS and Multiple Myeloma

MGUS is a common plasma-cell precursor condition, with prevalence increasing substantially with age. It affects approximately 4% of adults older than 50 years, whereas LC-MGUS represents a smaller subset of these cases [4,5]. Prevalence also varies between populations. In a large health-check screening cohort from Shenzhen, China, MGUS was identified in 1.17% of 49,220 participants, while a screening study of Black South African men aged over 30 years reported a prevalence of 8.03% [7,32]. Multiple myeloma is considerably less common than MGUS but represents an important malignant disorder within the same plasma-cell disease spectrum [19].

The Biology of the Ratio and the Origins of the Conventional Interval

The diagnostic logic of the κ/λ ratio is elegant precisely because it is indirect. Absolute free light chain concentrations rise in any state of increased immunoglobulin synthesis or reduced renal clearance, so neither isotype alone distinguishes a clone from inflammation or kidney disease. A clone, by contrast, produces a single isotype, and the ratio of the involved to the uninvolved chain therefore departs from unity in a way that polyclonal stimulation should not. This reasoning holds only if the two isotypes behave identically in every respect other than clonal production, and they do not [8,10,11,18].

Free κ circulates predominantly as a monomer of roughly 22.5 kDa, whereas free λ circulates predominantly as a dimer of roughly 45 kDa. The monomer is filtered at the glomerulus about three times faster than the dimer. In health, this differential is inconsequential because both are cleared efficiently, and the ratio settles near unity. When glomerular filtration fails, clearance shifts toward the slower reticuloendothelial route, κ accumulates disproportionately, and the ratio drifts upward for reasons that have nothing to do with clonality [8,12,18]. The same asymmetry that makes the ratio informative in myeloma makes it fragile in renal impairment.

Superimposed on this physiology is an analytical problem. Free light chain immunoassays detect epitopes that are hidden when the light chain is bound to a heavy chain, and different manufacturers raise antisera against different such epitopes, handle antigen excess differently, and calibrate against non-commutable materials. There is no international reference material for free light chains, and results are consequently not interchangeable between platforms [16,18-22]. Free light chains are also not inert waste products: they bind antigen, activate mast cells, and participate in inflammatory signaling, so their polyclonal elevation in chronic immune activation is a genuine biological phenomenon rather than analytical noise [34,35].

The conventional diagnostic interval of 0.26-1.65 may not adequately account for variation related to age and renal function [6,8], and subsequent population-based studies have supported the use of adjusted intervals in specific clinical populations [12-17]. Whether the interval survives that transfer is an empirical question, and the literature of the past decade provides substantial evidence that this transfer is imperfect.

Population Screening

The most informative estimate comes from the Iceland Screens, Treats or Prevents Multiple Myeloma (iStopMM) program, in which 41,882 participants aged 40 years or older with preserved kidney function were screened by electrophoresis, immunofixation and sFLC assay. Applying the standard interval, the ratio was abnormal in 3.7%, with κ abnormal in 17.5% and λ in 4.0%. Revised age-stratified 99% intervals of 0.44-2.16 for those under 70 years and 0.46-2.59 for those aged 70 years or older reduced the prevalence of LC-MGUS from 1.54% to 0.27%, an 82% fall. Over a median of 4.6 years of follow-up, none of the 1,006 individuals reclassified as normal progressed to a lymphoproliferative disorder [6], consistent with the removed diagnoses reflecting the limitations of the reference interval rather than early clonal disease, though a follow-up of this length cannot exclude indolent disease that had not yet progressed. External validation in a Danish nationwide clinical cohort subsequently reduced LC-MGUS classifications by 40% using the revised criteria; none of the reclassified individuals progressed to multiple myeloma or AL amyloidosis during follow-up, although two developed non-Hodgkin lymphoma [33].

Screening data from other settings reinforce the imperfect alignment between the ratio and clonality from the opposite direction. In 49,220 health-check attendees in Shenzhen, MGUS prevalence was 1.17% (95% CI 1.08-1.27), yet an abnormal free light chain ratio was present in only 31.1% of confirmed cases [7]. The ratio is therefore neither a specific nor a fully sensitive surrogate for MGUS in unselected populations.

Chronic Kidney Disease

Frequencies rise sharply once renal function is impaired. In the iStopMM CKD subgroup of 6,461 participants with an estimated glomerular filtration rate (eGFR) below 60 mL/min/1.73 m² and no evidence of monoclonality, the ratio lay outside the standard interval in 9.0% but outside the established renal interval of 0.37-3.10 in only 0.7%; κ was out of range in 60% and λ in 21% [8]. A Chinese multicenter cohort of 5,287 patients with non-dialysis CKD reproduced this pattern almost exactly: 10.5% abnormal by the standard interval, 0.3% by the renal interval, 0.5% by the iStopMM eGFR-banded intervals, and 0.9% by a locally derived interval. Crude LC-MGUS prevalence fell from 10.4% to 0.9%, and was κ-restricted in 99.8% of cases, a distribution that is itself difficult to reconcile with true clonal disease, in which λ-restricted clones are well represented [12].

Routine Laboratory Practice and Healthy Volunteers

The highest reported frequency arises not in a screened population but in ordinary clinical practice. Among 5,057 consecutive clinical samples without evidence of monoclonality on sFLC assessment and orthogonal immunofixation, a quarter (1,226/5,057) had a ratio outside 0.26-1.65. A locally derived interval of 0.67-2.13 reduced the rate of apparent abnormality to 8%, and the misclassification was not attributable to sex, age, kidney function, or antisera lot - implicating the provenance of the interval itself rather than any patient characteristic [13]. Even among rigorously screened healthy volunteers at three centers, 6-7% fell above the diagnostic range with both the prior and the reformulated κ calibrator, and the manufacturer's κ reference interval could not be verified with either [14]. A Spanish reference-interval study in 246 healthy blood donors derived a local diagnostic interval of 0.50-2.11, appreciably wider than the manufacturer's, and found classification discordant in 14.5% of MGUS and 16.3% of myeloma samples when the two intervals were compared [16]. The cohorts for which a proportion of abnormal ratios was actually reported are displayed in Figure 1 [6,8,12-14]. The reference intervals themselves manufacturer-recommended, age-stratified, renal, and locally derived are set side by side in Figure 2.

**Figure 1:**
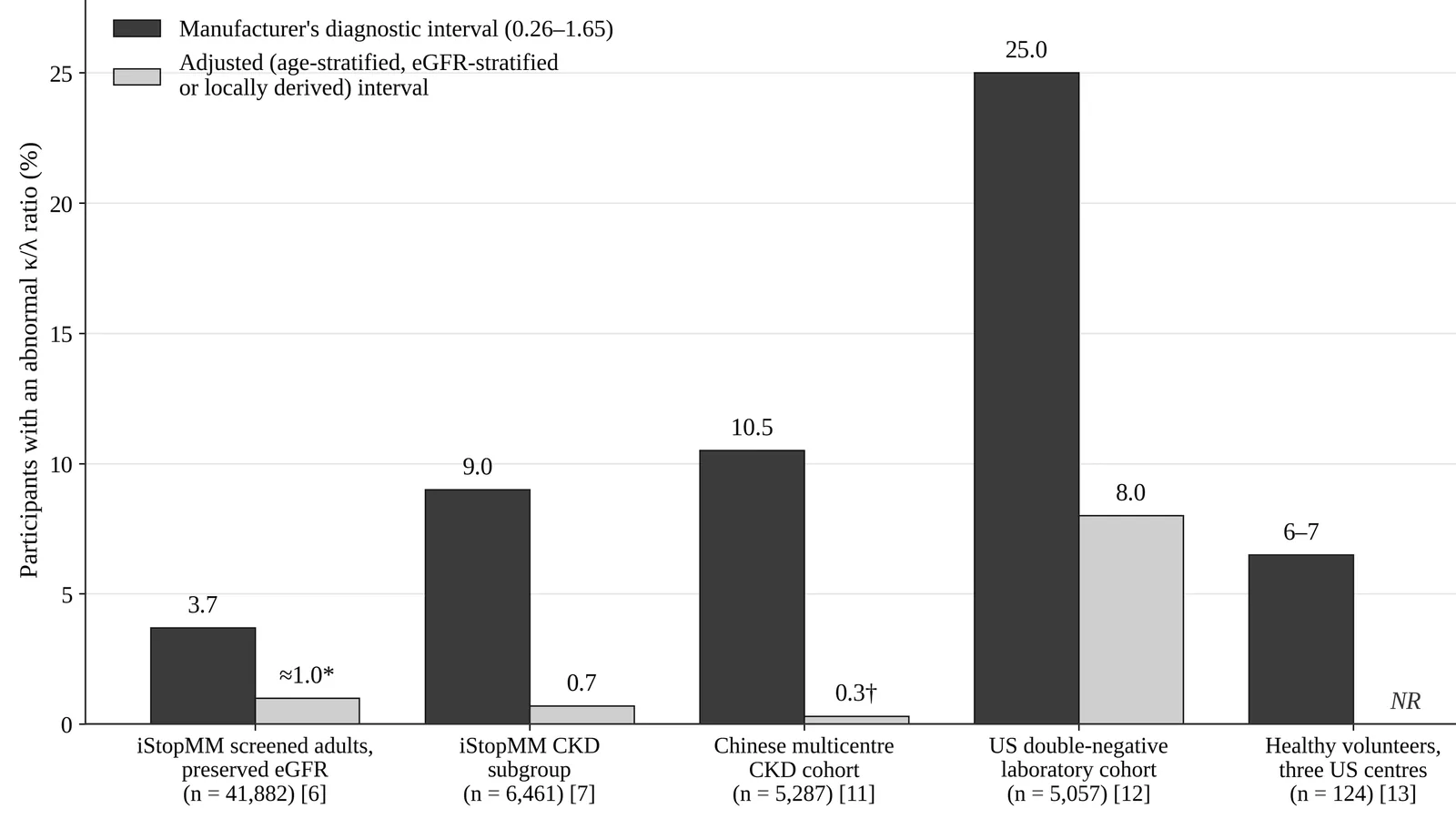
Frequency of an abnormal serum free light chain ratio in populations without clonal plasma cell disease *Approximately 1% by construction, because the revised iStopMM interval is a central 99% reference interval. †Value obtained with the renal interval (0.37-3.10); the corresponding figures were 0.5% with the iStopMM eGFR-banded intervals and 0.9% with a locally derived interval. NR, not reported Image created using Microsoft Excel (Microsoft® Corp., Redmond, WA). Image credit: Mudit Bhatnagar and Sri Krushna Prasad Mishra.

**Figure 2:**
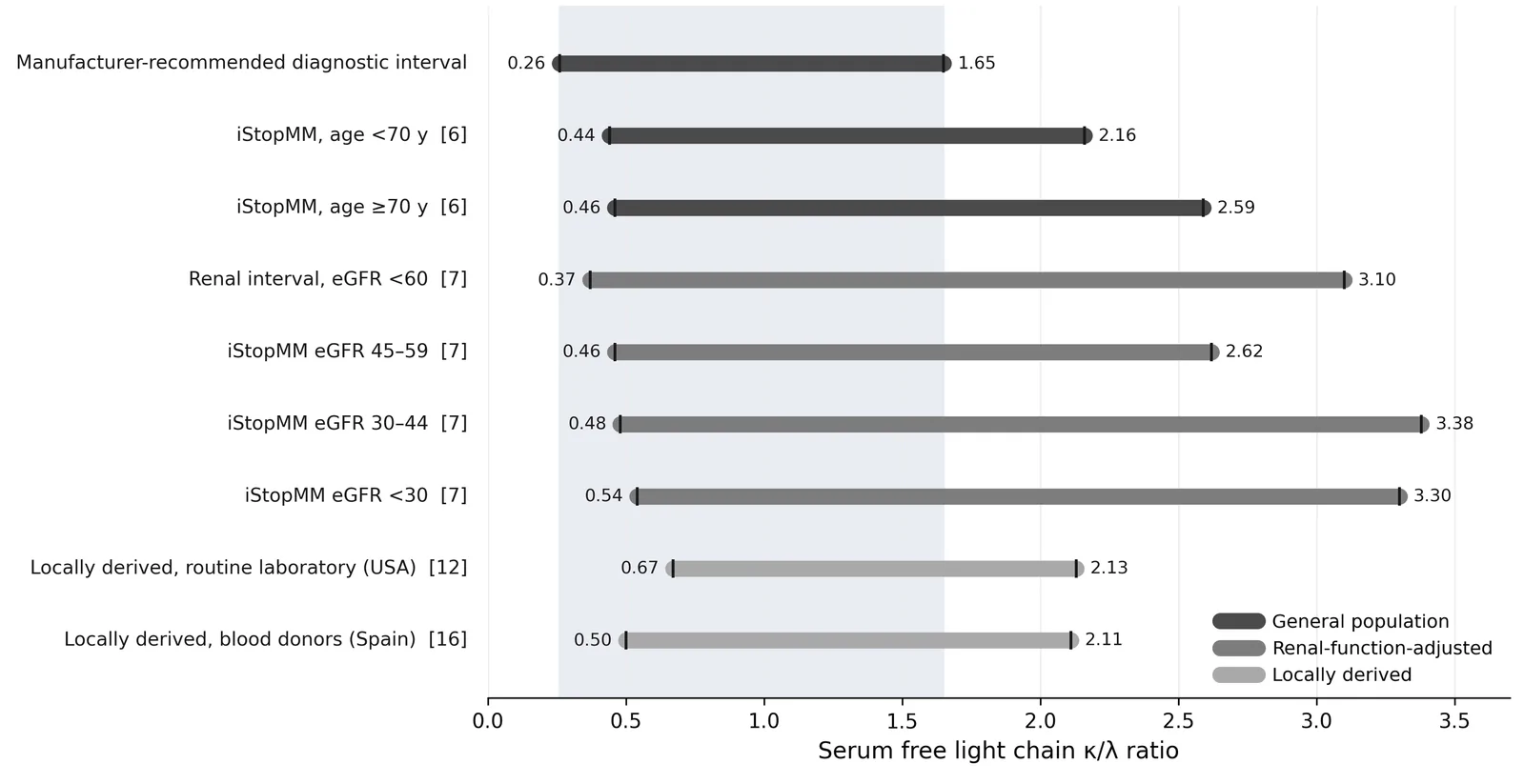
Reference and diagnostic intervals for the serum free light chain κ/λ ratio Each bar spans the lower to upper limit of the stated interval. Renal-function-adjusted and locally derived intervals are consistently wider than, and shifted above, the manufacturer’s interval (shaded band), so a value read as abnormal on the manufacturer’s interval may fall within an interval appropriate to the individual’s age or renal function. eGFR is expressed in mL/min/1.73 m². eGFR, estimated glomerular filtration rate; iStopMM, Iceland Screens, Treats, or Prevents Multiple Myeloma Image created using Microsoft Excel (Microsoft® Corp., Redmond, WA). Image credit: Ambili Remesh and Akhil Jose.

Determinants of an abnormal ratio

Reduced Glomerular Filtration

Renal function is among the strongest and most consistently observed determinants. With the Freelite assay, the ratio rises progressively as eGFR falls, following the differential-filtration mechanism outlined above. The effect is large enough that eGFR-banded intervals of 0.46-2.62, 0.48-3.38 and 0.54-3.30 have been proposed for eGFR strata of 45-59, 30-44 and below 30 mL/min/1.73 m² respectively [8]. Refined eGFR-dependent cut-offs materially improved the exclusion of monoclonal gammopathy in a multinational cardiac amyloidosis cohort of 3,354 patients, in which non-biopsy criteria for transthyretin cardiomyopathy achieved 97% specificity (95% CI 91%-99%) [23]. A single renally robust ratio interval, and a principal-component-based metric combining both isotypes, have each been shown to lower the rate of apparent abnormality across the full spectrum of renal function without loss of sensitivity for monoclonal gammopathy [15]. The differential renal handling of the two isotypes that produces this gradient is shown schematically in Figure 3.

**Figure 3:**
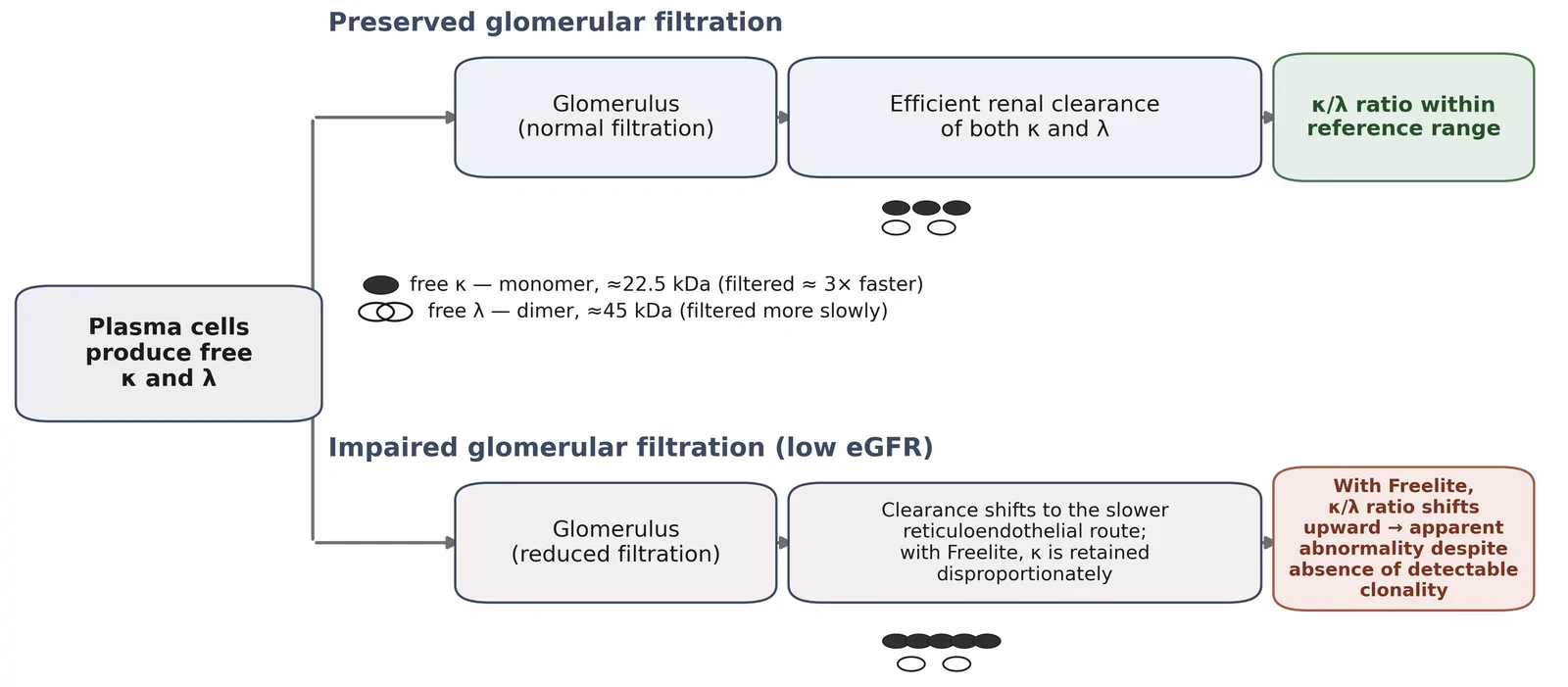
Mechanistic basis for an increased serum free light chain κ/λ ratio with declining renal function Plasma cells produce free κ and λ light chains. With preserved glomerular filtration, both light chains undergo efficient renal clearance, and the κ/λ ratio generally remains within the reference range. With impaired glomerular filtration, clearance shifts toward the slower reticuloendothelial pathway, with greater retention of κ relative to λ, resulting in an upward shift in the κ/λ ratio. With the Freelite assay, this may produce an apparent abnormal ratio in the absence of detectable monoclonality. Based on References [8,12,15,18,19,20,21,22]. eGFR, estimated glomerular filtration rate; κ, kappa; λ, lambda Image created using Canva (Canva Inc., San Francisco, CA). Image credit: Mohammed Mounuddin, Akhil Jose, and Gaurav Borse.

Assay Platform and Calibration

Platform effects are the second major determinant, and they run in opposite directions between manufacturers. In 226 identical sera from patients with CKD stages 2-5, the median ratio was 1.50 (range 0.66-3.45) by Freelite but 0.63 (95% range 0.34-1.01) by Kloneus [19]. The Freelite ratio correlates inversely with eGFR, whereas the N Latex ratio correlates positively [18], and the N Latex ratio remains within the healthy reference range across the full range of renal function, while the Freelite ratio does not, tracking β₂-microglobulin closely instead [22]. The Sebia assay behaves differently again and requires its own renal ranges, showing 82% concordance with Freelite and raised κ and λ in CKD [20,21]. A head-to-head comparison in 260 sera found that at eGFR ≥60 the ratio met the manufacturer's claim in 91% of Freelite and 95% of Diazyme measurements, but that the two diverged in opposite directions as eGFR fell [16].

Analytical drift compounds the problem. Upward drift in κ measurement has been documented and was not corrected by a reformulated calibrator, with 6-7% of healthy participants remaining above the diagnostic range on either calibrator lot [13,14]. Analytical validation work on polyclonal free light chain assays points the same way: platforms agree more closely on the summed free light chains than on the individual isotypes, and therefore agree least on the ratio [36]. The practical consequence is that an assay change can convert an abnormal ratio into a normal one in the same patient overnight.

Advancing Age

Age is associated with a modest but measurable widening of the ratio interval, with the upper limit of the ratio rising from 2.16 below 70 years to 2.59 at 70 years and above, and the κ and λ upper limits rising in parallel [6]. The likely mechanism combines age-related decline in glomerular filtration with immunosenescence and age-associated oligoclonal B-cell expansion. Since the tested population skews old, an interval derived from young donors is systematically too narrow at the point of use.

Polyclonal B-cell Activation in Autoimmune, Infectious and Critical Illness

Polyclonal activation is an important non-renal biological determinant, and it is systematically underappreciated at the bedside. In a European cross-sectional study of 1,979 patients with systemic autoimmune disease and 614 healthy controls, total free light chains were raised in 28.4% of patients with a preserved κ/λ ratio, and the elevation tracked disease activity and the type I interferon signature rather than kidney function [29]. In a smaller Italian series spanning rheumatoid arthritis, systemic lupus erythematosus, antiphospholipid syndrome, primary Sjögren syndrome and hepatitis C-associated cryoglobulinaemia, κ rose more than λ across every group, producing a mean ratio of approximately 1.6 that is, sitting at the very edge of the standard upper limit [30]. A patient population clustered at the threshold requires only a modest additional perturbation from renal impairment or assay bias to be pushed across it.

Acute illness produces the same shift more abruptly. On the first day of intensive care, 75.3% of 101 septic patients without immune comorbidity had a high κ and 46.5% a high λ, in proportion to the frequency of dual κ/λ-expressing CD19⁺ B cells, which were more than three times as common as in healthy controls [31]. Combined free light chains have also been reported to predict death after cardiac-surgery-associated acute kidney injury, although inflammation and renal dysfunction are inseparably confounded in that setting [37]. A caution is warranted here. The screened-cohort literature indicates that this polyclonal shift should not be mistaken for an increased burden of true clonal disease. In iStopMM, autoimmune disease was not associated with screen-detected MGUS (prevalence ratio 1.05, 95% CI 0.97-1.15), although it was strongly associated with clinically diagnosed MGUS (prevalence ratio 2.11, 95% CI 1.64-2.70) - illustrating the potential for ascertainment bias [38,39]. Earlier studies conducted in clinical rather than population-screened cohorts have reported associations between MGUS and autoimmune or infectious disease; however, differences in case ascertainment and referral patterns may limit the generalizability of these findings [40].

Ancestry, Environment, and Host Factors

Background differences matter for interpretation because they set the baseline rate of genuine clonal disease against which a non-clonal abnormal ratio must be judged, and because some of the same exposures also raise background polyclonal activation; in this sense, the material below is contextual rather than a direct determinant of the ratio itself. MGUS prevalence is higher in Black South African men than in White men in Olmsted County. Current smoking was independently associated with MGUS (adjusted OR 4.49, 95% CI 1.63-14.56), while the apparent association with HIV status was no longer statistically significant after adjustment for BMI and cigarette use [32]. LC-MGUS is markedly more common in World Trade Center-exposed firefighters than in the Olmsted reference population [41], and abnormal ratios cluster in relatives from high-risk chronic lymphocytic leukemia pedigrees, consistent with a heritable component to immunoglobulin biomarker variation [42]. Obesity accelerates progression from MGUS to myeloma rather than its incidence, with cumulative excess body mass index after diagnosis carrying incremental risk [43,44], and prior cancer carries only a modest, transient excess risk [45]. None of these factors has been shown to shift the ratio directly in a non-clonal individual; their relevance is contextual, shaping the pre-test probability of true clonality in the population being tested.

Transience of the Abnormality

An abnormal ratio is frequently not reproducible. In the population-based Heinz Nixdorf Recall study, 17 of the 31 screen-detected LC-MGUS cases that had repeat samples (55%) could not be confirmed on retesting, a proportion based on only 31 of the 75 identified cases, so the precise figure is uncertain, and none of the 75 cases progressed over a median of 11.5 years [26]. Analytical imprecision superimposed on transient polyclonal or oligoclonal expansion offers a sufficient explanation. This single observation carries as much practical weight as any of the interval revisions, because it applies to every laboratory irrespective of which interval it reports against. The determinants, their direction and magnitude, and the proposed mechanisms are set out in Table 2. Figure 4 groups the principal non-clonal determinants into the three domains renal and physiological, analytical, and immunological and host-related used throughout this review.

**Table 2:** Determinants of an abnormal serum free light chain ratio in the absence of clonal plasma cell disease Each row lists a factor reported to shift the serum free light chain κ/λ ratio outside its reference interval in the absence of a clonal plasma cell population, the direction and approximate magnitude of that shift as reported in the literature, and the biological or analytical mechanism proposed to explain it. AKI, acute kidney injury; BAFF, B-cell activating factor; CKD, chronic kidney disease; eGFR, estimated glomerular filtration rate; FLC, free light chain; ICU, intensive care unit

Determinant	Effect on FLC concentrations and κ/λ ratio	Mechanism	References
Reduced glomerular filtration	With Freelite, the κ/λ ratio rises progressively as eGFR falls. An abnormal ratio was found in 9.0-10.5% of non-dialysis CKD cohorts using the standard interval, compared with 0.3-0.7% using renal intervals.	Loss of the dominant renal clearance route. κ is cleared more rapidly than λ under normal conditions; with reduced filtration, clearance shifts toward the reticuloendothelial system and κ may accumulate disproportionately.	[8,12,15,18-22]
Assay platform	Substantial differences in the measured κ/λ ratio occur between platforms. In identical CKD sera, median ratios differed between Freelite and Kloneus; Freelite and N Latex also show different relationships with eGFR.	Differences in assay antibodies, calibration, analytical characteristics, and platform-specific measurement of κ and λ.	[16,18-21]
Calibrator/lot drift	Upward drift in κ measurement has been documented; a reformulated κ calibrator did not fully restore the claimed reference interval, with 6-7% of healthy volunteers remaining above the manufacturer's ratio interval.	Changes in calibration and analytical performance can alter measured κ concentrations and consequently the κ/λ ratio.	[13,14]
Provenance of reference interval	The manufacturer's interval of 0.26-1.65 classified 3.7-25% of individuals as abnormal across different cohorts; locally derived or population-specific intervals substantially reduced the proportion classified as abnormal.	Reference intervals derived from different populations and analytical platforms may not be interchangeable.	[7,8,13,17]
Advancing age	The upper limit of the ratio increased from 2.16 in individuals aged <70 years to 2.59 in those aged ≥70 years, with κ and λ concentrations also increasing with age.	Age-related changes in renal function and immunoglobulin/light-chain biology may broaden the distribution of FLC values.	[8]
Polyclonal immune activation in autoimmune disease	Both κ and λ concentrations may increase, while the κ/λ ratio often remains within or near the expected range. Mild ratio abnormalities may occur in some conditions.	Increased polyclonal immunoglobulin synthesis raises both free light-chain concentrations; disproportionate expansion of one light-chain population may occasionally shift the ratio.	[27,29,30,34,35]
Infection/sepsis/critical illness	κ and λ concentrations are frequently elevated; increased summed FLC reflects polyclonal activation and/or impaired renal clearance and should not be equated with an abnormal κ/λ ratio.	Acute polyclonal immunoglobulin synthesis, expansion of dual κ/λ-expressing B cells, and concurrent acute kidney injury may contribute.	[31,37]

**Figure 4:**
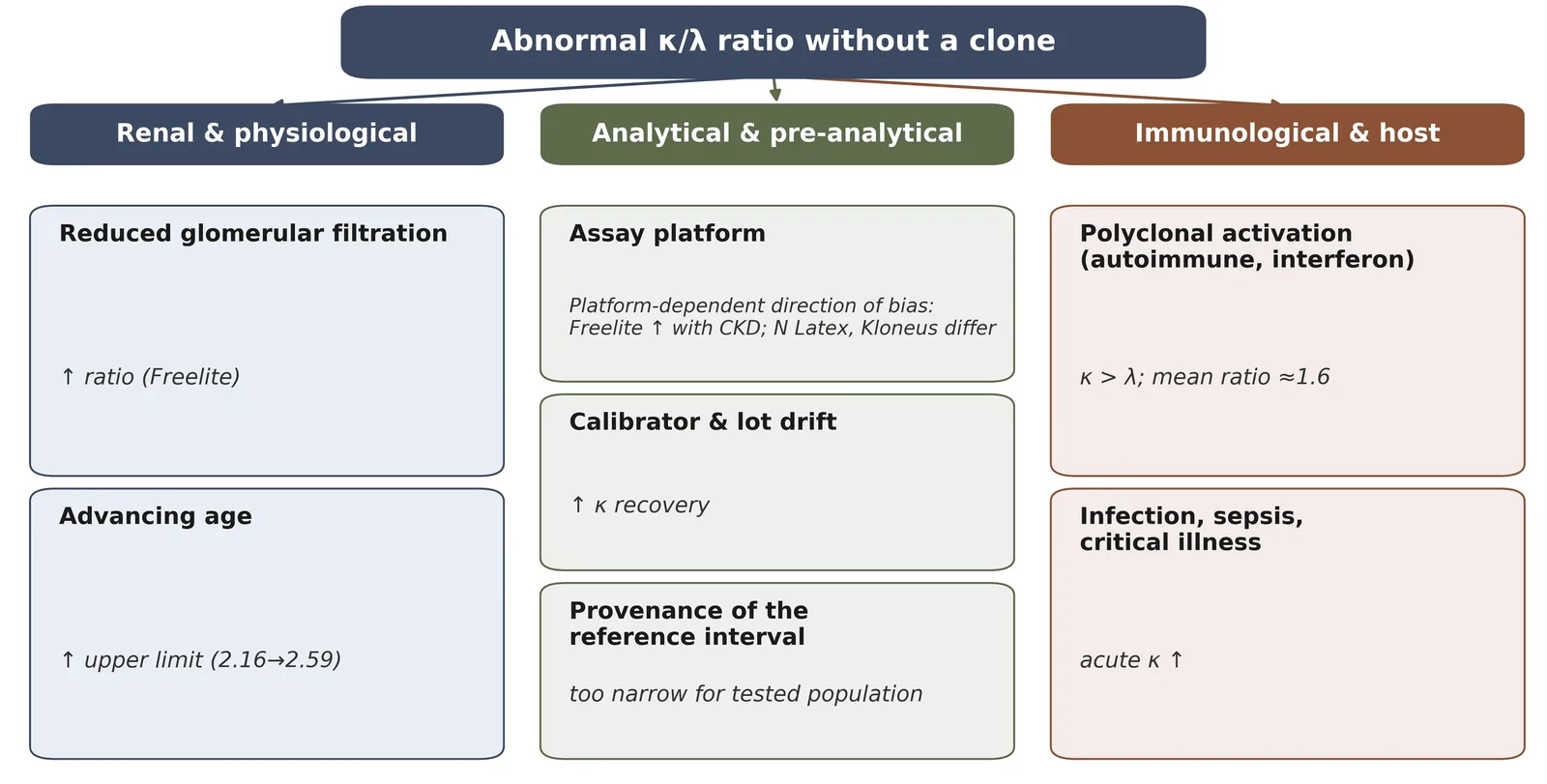
A framework for the principal non-clonal determinants of an abnormal κ/λ ratio An abnormal κ/λ ratio may occur in the absence of detectable monoclonality through several overlapping mechanisms. These include renal and physiological factors, such as reduced glomerular filtration and advancing age; analytical and pre-analytical factors, including assay-platform differences, calibrator or lot-related variation, and limitations of the reference interval; and immunological and host factors, including polyclonal immune activation and infection, sepsis, or critical illness. The direction and magnitude of the effect may vary according to the underlying mechanism and assay platform. κ, kappa; λ, lambda; CKD, chronic kidney disease. Based on references [8,13-22,27,29-35]. Image created using Canva (Canva Inc., San Francisco, CA). Image credit: Akhil Jose, Mudit Bhatnagar, and Mohammed Mounuddin.

The Matter of a Non-clonal Abnormal Ratio

An abnormal or elevated free light chain result in the absence of a clone is not always inert. Among 15,630 Olmsted County residents without MGUS at baseline, the highest decile of summed polyclonal free light chains carried a 2.6-fold increased risk (95% CI 1.8-3.7) of subsequently developing a monoclonal gammopathy over a median 18.1 years, suggesting that a chronic inflammatory state may precede clonal emergence [27]. In CKD, summed free light chains above 43.3 mg/L independently predicted both death (hazard ratio 1.45, 95% CI 1.14-1.85) and progression to end-stage renal disease (hazard ratio 3.25, 95% CI 1.32-7.99) across 3,912 participants in five prospective cohorts [28].

These findings concern the summed concentration rather than the ratio, and the distinction matters. The evidence supports a model in which chronic antigenic stimulation and host factors raise the probability of both polyclonal activation and eventual clonal emergence, while the ratio at any single time point remains a poor marker of either. Polyclonal burden may carry prognostic information; a solitary skewed ratio in a non-clonal individual, by contrast, is more often an artefact of interval, platform or kidney than a signal of anything.

Implications for Laboratory Reporting and Clinical Practice

Three practical conclusions follow from the literature reviewed. First, laboratories may wish to move beyond reporting the ratio against a single manufacturer-recommended range alone. Reporting against age- and eGFR-appropriate reference intervals, verified locally where feasible and accompanied by an interpretive comment, is supported by consistent, although not yet pooled, evidence. These approaches have reduced light-chain MGUS diagnoses or apparent abnormality rates several-fold across screening, CKD, and routine laboratory cohorts [6,8,12,13]. The 82% reduction seen in the Icelandic screening cohort was followed by a 40% reduction in LC-MGUS classifications in an independent Danish clinical validation cohort, again without progression to multiple myeloma or AL amyloidosis among reclassified individuals [33]. These findings are clinically relevant because they can substantially alter the number of individuals classified as having a precursor disorder.

Second, where an isolated abnormal ratio would alter management, confirmation on a fresh sample should be considered, with orthogonal testing where appropriate. Immunotyping by capillary electrophoresis performs equivalently to immunofixation for intact monoclonal proteins [46], urine free light chain quantification has limited utility for first-time urine screening and does not reliably exclude a monoclonal light chain [47], and mass spectrometry now offers the most sensitive route: MASS-FIX detected a monoclonal protein in every sample with a measurable electrophoretic spike across 19,523 patients and outperformed immunofixation in confirming abnormal free light chain ratios [48,49]. Orthogonal testing also guards against the opposite error, since a normal ratio persisted in 16% (14/85) of samples in which free λ was demonstrable by immunofixation [13]. Where the question is whether a small clone is causing kidney disease, prediction tools that combine the involved-to-uninvolved ratio with proteinuria, complement, and hematuria can help decide whether a biopsy is warranted [11], and validated models can spare bone marrow sampling in low-risk MGUS [50].

Third, assay manufacturers and standardization bodies should prioritize a commutable international reference material. The absence of one remains the central unsolved problem, and the finding that a reformulated κ calibrator failed to restore the manufacturer's claimed interval suggests it will not be solved by recalibration alone [14].

Gaps in the Evidence and Directions for Future Work

Several gaps qualify the conclusions drawn here. Heterogeneity in assay platform, reference interval, case mix, and the operational definition of an abnormal ratio means that the frequencies presented are descriptive and should not be pooled or read as a single estimate. Most of the available evidence comes from the Freelite assay, which limits transfer to laboratories running other platforms, and the studies that compared platforms directly were generally small. Very few cohorts originate from South Asia, Latin America, or the Middle East, so ancestry- and region-specific intervals remain largely undefined, a conspicuous gap given the documented differences in background prevalence by ancestry and exposure. Several reference-interval studies recruited blood donors or volunteers younger and healthier than the tested population, which would tend to make the derived intervals too narrow, and amyloidosis cohorts are subject to referral bias by design. Longitudinal follow-up was available in only a minority of reports, so the natural history of a non-clonal abnormal ratio is characterized less securely than its prevalence. Restriction of the literature surveyed to English-language publications and to a ten-year window may also have omitted relevant work. Future work should establish region- and ancestry-specific intervals in under-represented populations, extend eGFR-banded intervals to non-Freelite platforms, and follow non-clonal abnormal ratios prospectively to define their long-term significance.

## Conclusions

An abnormal sFLC ratio can occur in individuals without detectable clonal plasma cell disease, particularly when renal impairment, age, assay-related variation, or polyclonal immune activation is present. Interpretation should therefore account for the patient's renal function, age, clinical context, and assay platform rather than relying on the conventional reference interval alone. Age- and renal-function-adjusted reference intervals may improve diagnostic specificity, while repeat and, when appropriate, orthogonal testing can help distinguish persistent clinically relevant abnormalities from isolated or non-clonal findings. Further prospective studies are needed to establish platform-specific and population-specific reference intervals and to define the long-term significance of persistent non-clonal abnormalities.
